# Unlike Zika, Chikungunya virus interferes in the viability of *Aedes aegypti* eggs, regardless of females’ age

**DOI:** 10.1038/s41598-020-70367-6

**Published:** 2020-08-12

**Authors:** Maria Eduarda Barreto Resck, Karine Pedreira Padilha, Aline Possati Cupolillo, Octávio A. C. Talyuli, Anielly Ferreira-de-Brito, Ricardo Lourenço-de-Oliveira, Luana Cristina Farnesi, Rafaela Vieira Bruno

**Affiliations:** 1 Laboratório de Biologia Molecular de Insetos, Instituto Oswaldo Cruz, Fundação Oswaldo Cruz - Fiocruz, Rio de Janeiro, RJ Brazil; 2 Laboratório de Bioquímica de Insetos Hematófagos, Instituto de Bioquímica Médica Leopoldo de Meis, Universidade Federal Do Rio de Janeiro, Rio de Janeiro, RJ Brazil; 3 Laboratório de Mosquitos Transmissores de Hematozoários, Instituto Oswaldo Cruz, Fundação Oswaldo Cruz - Fiocruz, Rio de Janeiro, RJ Brazil; 4 Instituto Nacional de Ciência e Tecnologia em Entomologia Médica, Conselho Nacional de Desenvolvimento Científico e Tecnológico, Rio de Janeiro, Brazil

**Keywords:** Viral infection, Infectious diseases

## Abstract

Chikungunya and Zika are arboviruses transmitted by the mosquito *Aedes aegypti*. Mosquito fecundity and egg viability are important parameters of vectorial capacity. Here we aim to understand, comparatively, the effects of Chikungunya virus (CHIKV) and Zika virus (ZIKV) infections on the fecundity and fertility of young and old *Aedes aegypti* females. Using artificial infection blood feeding experiments we observed that both CHIKV and ZIKV do not alter the number of eggs laid when compared to uninfected females, although the egg fertility significantly decreases in both young and old CHIKV-infected females. There is an upward trend of null females (infertile females) from 2.1% in young to 6.8% in old ZIKV-infected females. Together, our data revealed that CHIKV and ZIKV affects differently *Ae. aegypti* physiology, that may be related to different viral spread in nature.

## Introduction

Chikungunya virus (CHIKV), a member of the *Togaviridae* family, *Alphavirus* genus, was first isolated in 1952 in Tanzania^1^. Emerging and reemerging outbreaks have occurred since its discovery in several regions in Africa, Asia, Indian Ocean islands and Mediterranean areas in Europe^2,3^. In Brazil, the first Chikungunya autochthonous cases occurred in 2014^4^. The virus spread throughout the country^5^, being in co-circulation with dengue (DENV) and Zika (ZIKV) viruses during this period^6^. Zika, a virus belongs to *Flaviviridae* family, *Flavivirus* genus, was first isolated in 1947^7^. For 60 years, only sporadic Zika cases were reported in humans, however in 2007 an outbreak occurred in Yap Island, Micronesia^8^. Subsequently, ZIKV expanded throughout the Pacific islands and reached the Americas in 2015, turning into an epidemic in Brazil. It is currently considered a new public health threat^9–11^.

Both arboviruses (Zika and Chikungunya) are transmitted by the bite of mosquitoes of genus *Aedes*^7,12,13^. Among them, the *Aedes aegypti* is the main vector in the urban transmission cycle^14–16^. This domestic and anthropophilic species is anautogenous (the females need blood supply for eggs maturation)^17^. After one or more blood feedings, egg maturation occurs in about 3 or 4 days. Each period between blood feeding and egg laying is called gonotrophic cycle (GC)^17–19^. An *Ae. aegypti* female is able to lay approximately 100 eggs per GC^20^. The ingestion of more than one blood meal by mosquito females within a single gonotrophic cycle is called gonotrophic discordance and can occur in *Aedes aegypti* mosquitoes. This feature is involved in vectorial capacity and is very important for the transmission of viruses^21–23^.

Comparatively, the biology of the egg phase is less explored than other in the mosquito life cycle. The *Ae. aegypti* eggs can survive for long periods in dry conditions at the end of their embryonic development^17,19,24–26^. This important feature is related to ecological issues such as dormancy, that enables the embryo to survive drought periods^19^. Furthermore, egg resistance to desiccation (ERD) facilitates passive dispersal to new locations being important to epidemiologic features of arboviruses that has *Ae. aegypti* as the main vector^27^.

Distinct arbovirus may replicate in different velocities and consequently the time between taking a viremic blood meal and shedding virus into saliva (extrinsic incubation period, EIP) vary. CHIKV reaches the *Aedes* salivary glands faster than DENV2, which directly affects the potential of viral spread^28^. During the EIP, virus replicate in the midgut and in several secondary mosquito tissues following disseminate in the hemocoel prior to be shed into saliva. Thus, arbovirus can interfere in female biology and consequently in their physiology such as fecundity and fertility^29–31^. Hence, the analysis of number of eggs laid by an infected mosquito (fecundity) and their viability (fertility) are also key parameters to comprehend the vectorial capacity.

Our previous studies have shown that ZIKV infection can modulate *Ae. aegypti* females’ locomotor activity but did not change significantly the fecundity and fertility neither in the second or third GCs when the female egg laying is clustered^30^. Here we refined our methodology focusing on understand, comparatively, the effect of infection by CHIKV and ZIKV in fecundity and fertility of *Ae. aegypti* females from different ages, analyzed at distinct GC.

## Results

### CHIKV and ZIKV infection do not change the fecundity

**Figure 1:**
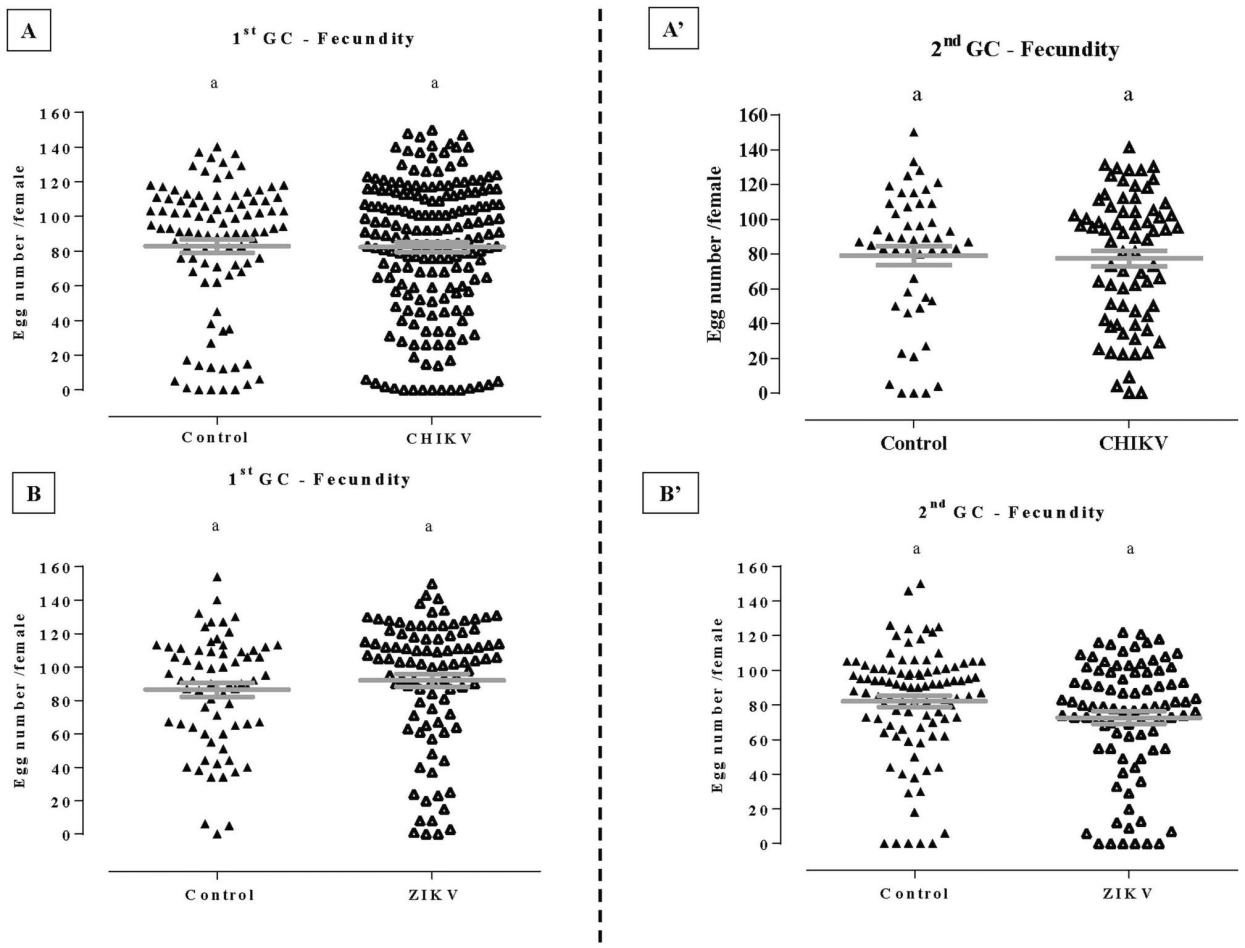
Effect of Chikungunya (CHIKV) and Zika (ZIKV) viruses infections on first gonotrophic cycle (GC) (**A**,**B**) and second GC (**Aʹ**,**Bʹ**) fecundity of *Aedes aegypti* females. The significance is represented by *p* < 0.05 obtained by using the non-parametric Mann–Whitney test. Bars represent mean and ± se of independent experiments.

We analyzed whether CHIKV and ZIKV infection influenced in the fecundity in females of different ages following the first or second GCs. Infection rate of randomly chosen samples of mosquitoes orally challenged with viruses was 80% for CHIKV infection and 89.5% for ZIKV infection. . We observed that the number of eggs did not change in any of the tested settings for CHIKV infected (7-days-old or 14-days-old females; *p* = 0.88 and *p* = 0.96, respectively) and non-infected mosquitoes (Fig. 1A,Aʹ). The same was observed when females were infected with ZIKV, where no significant differences were observed in fecundity, independent of the females’ age (*p* = 0.09 and *p* = 0.0542, respectively) (Fig. 1B,Bʹ).

### CHIKV infection affects females’ fertility, dissimilar from ZIKV infection

**Figure 2:**
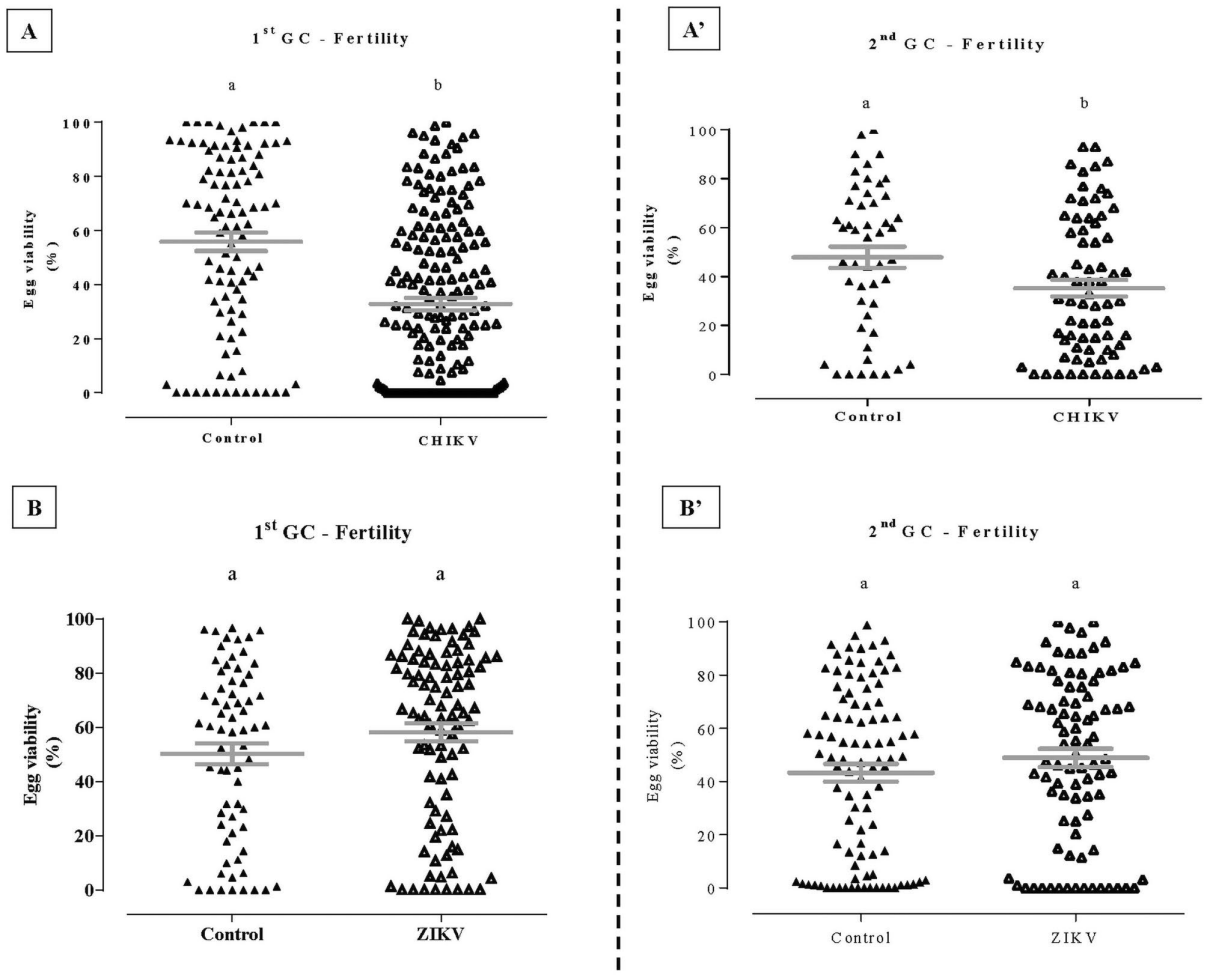
Effect of Chikungunya (CHIKV) and Zika (ZIKV) viruses infections on first GC (**A**,**B**) and second GC (**Aʹ**,**Bʹ**) fertility of *Aedes aegypti* females. The significance is represented by *p* < 0.05 obtained by using the non-parametric Mann–Whitney test. Bars represent mean and ± se of independent experiments.

As opposed to the fecundity, the fertility (the quantity of egg hatching) was affected by CHIKV infection. When females were younger (infectious blood meal taken in the 1^st^ GC), the average of fertility in non-infected l was 55.77 (± 3.43) compared to 32.73 (± 2.27) in CHIKV infected females. In addition, when females were older (infectious feeding was performed in the 2^nd^ GC), the average of the control group was 47.88 (± 4.31) and decreased to 35.22 (± 3.37) in the treated group (Fig. 2A,Aʹ) (*p* < 0.05 in both cases).

We evaluated the influence of ZIKV infection in *Ae. aegypti*’s fertility, in the same conditions of CHIKV (females were blood fed in different ages/GCs). We did not observe any significant difference for ZIKV infected and non-infected *Ae. aegypti* females’ fertility (Fig. 2B,Bʹ; *p* = 0.09 and *p* = 0.35, respectively).

### Fecundity and fertility lose correlation when females are infected with CHIKV

**Figure 3:**
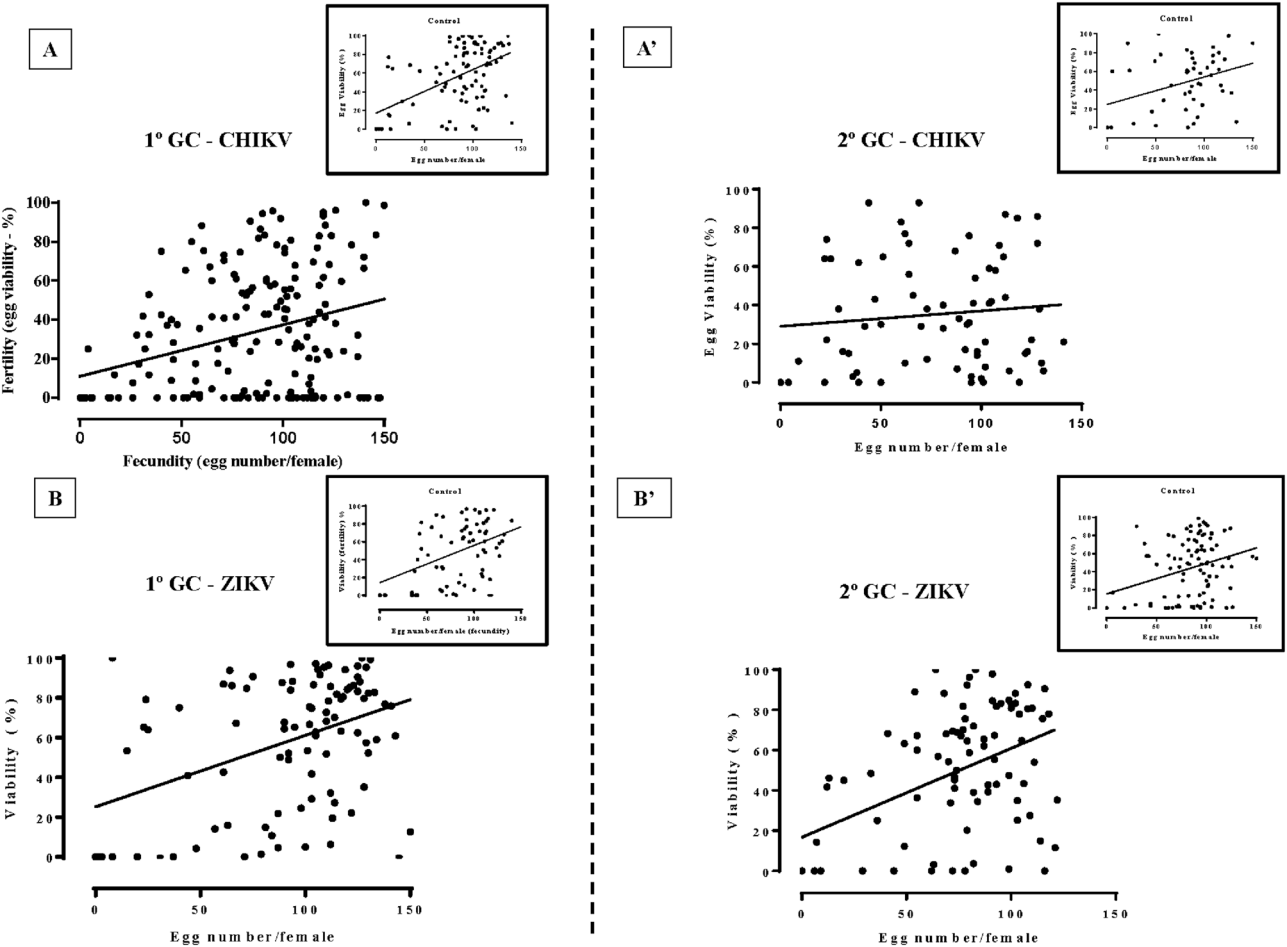
Correlation between fecundity and fertility of *Aedes aegypti* females uninfected and infected with Chikungunya or Zika virus. (**A**): CHIKV—1º gonotrophic cycle (GC); (**Aʹ**): CHIKV—2º gonotrophic cycle (GC); (**B**): ZIKV—1º gonotrophic cycle (GC); (**Bʹ**): ZIKV—2º gonotrophic cycle (GC). All boxes shows controls of each conditions. Analysis was made for Correlation of Spearman, the R number of each correlation is described in the text.

We analyzed the correlation between fecundity and fertility in the first and second GCs of females infected with CHIKV or ZIKV. In the former, we can observe that the 7-days-old and 14-days-old females from the control group showed a positive correlation (Fig. 3A,Aʹ boxes; r = 0.45 and r = 0.33, respectively; *p* < 0.0001, *p* = 0.02, respectively). On the other hand, correlation data from females infected with CHIKV are different when feeding occurred in the first (young females) or second GC (old females). In the first cycle, the positive correlation observed in the control is maintained (r = 0.30; *p* < 0.0001), however, in the second GC this correlation is lost (r = 0.11, *p* = 0.35) (Fig. 3A,Aʹ).

Analyzing the correlation between fecundity and fertility in ZIKV young and old females, we observed a positive linear relationship in the control group (Fig. 3B,Bʹ boxes; r =  0.42; r = 0.27, respectively and *p* = 0.004, *p* = 0.0065, respectively). When analyzing infected *Ae. aegypti* females’ eggs throughout the ages, this positive correlation is still maintained (Fig. 3: B,Bʹ; r = 0.38; r = 0.40, respectively and *p* = 0.0002, *p* = 0.0002, respectively).

### Oviposition efficiency for CHIKV and ZIKV

We considered as oviposition efficiency the percentage of infected females (with CHIKV or ZIKV) that presented an oviposition value equal to or greater than the median value of control females. In the CHIKV infection, we obtained 49.5% of efficient females when feeding was performed in the first GC and 52.8% when it occurred in the second one. For ZIKV infection, oviposition efficiency was of 64.9% and 35.2% for first and second GCs, respectively. Regarding the number of nulls, that is, females that did not lay eggs, we obtained for CHIKV infection 3.8% and 2.7% when virus challenge was performed in the first or second GC, respectively. When analyzing the number of null females for ZIKV infection, the percentage increased from 2.1% in the first to 6.8% in the second GC (Table 1). In the case of ZIKV null females, we observed an increase of infertility in ZIKV infected females in second GC.

**Table 1:** Oviposition efficiency of *Ae. aegypti* females infected with CHIKV or ZIKV.

	N	Median	% of efficient females^a^	% of null females^b^
**1st gonotrophic cycle**				
CHIKV	186	91	49.5	3.8
ZIKV	97	92	64.9	2.1
**2nd gonotrophic cycle**				
CHIKV	72	87	52.8	2.7
ZIKV	88	91	35.2	6.8

*N* number of females.

^a^Efficiency was measured according to the control median.

^b^Females that did not lay eggs were considered null.

## Discussion

Vectorial capacity (VC), critical for arboviruses transmission, is the predictable value through a mathematical formula that considers beyond environmental conditions, intraspecific physiological and behavioral parameters, those that will be important to arboviruses transmission in nature^32,33^. The VC contemplates mainly biting behavior (frequency of host contact for blood feeding), mosquito-vector survivorship and population density^32–36^. Population density is very dependent on mosquito fecundity and fertility. *Aedes aegypti* behavior and physiology as well as arbovirus-mosquito interactions are thoroughly studied topics, but additional studies are needed in order to endorse the development of new tools to mosquito control actions.

There are different methods described in the literature to study the fertility of *Ae. aegypti* females^24,37,38^. Here, we used the methodology called synchronized posture that is successful in forcing females *Ae. aegypti* to lay their eggs in short time intervals^24,39^. The calculation of the oviposition time of 90 min was based on a comparison of the average number of eggs per female from previous studies in our group with longer time intervals^37^. In that paper, we showed a female laid an average number of 82 eggs in synchronized postures that lasted 6 h, while we present in this study an average number of 86 eggs per female in postures lasting 90 min. Therefore, we optimized the bioassay and achieved success in a shorter time.

In 2018, Padilha and collaborators^30^ tested the effects of ZIKV in fecundity and fertility in clusters of infected and non-infected *Ae. aegypti.* Here we aimed for a more accurate evaluation, where eggs laid by individual females were analyzed for additional parameters such as oviposition efficiency and number of null females, and tested correlation between fecundity and fertility.

We examined whether CHIKV or ZIKV infection could affect the number of eggs laid per females and hatching. We noted that, regardless of female age, CHIKV infection does not alter fecundity, but had significant influence on fertility. It is important to report that our results are different from those observed by Sirisena et al.^31^, when using a different methodology from ours. They performed their experiments with different mosquito and virus strains. The oviposition was carried out with females in group and during three GCs, where infective blood feeding occurred only in the first GC. Sirisena et al.^31^ found that CHIKV infection caused the group of females to lay fewer eggs than the control in the first and third GCs but not in the second one; the authors did not test for fertility of individual females.

In relation to ZIKV, despite using different methodologies, our data corroborate those of Padilha et al.^30^, where ZIKV infection does not cause damage to the overall fertility and viability of *Ae. aegypti*, but with a decreasing tendency (*p* = 0.0542) of egg numbers laid by older females. On the other hand, Petersen et al.^38^ considered the first three clutches of eggs individually laid by young (7 days), mature (14 days) and old (21 days) *Ae. aegypti* females, analyzing two aspects of fecundity: the oviposition success and clutch size. These authors observed that ZIKV infected mosquitoes laid fewer eggs than the uninfected ones and the egg production was affected by the age of feeding, once younger females laid more eggs than the older ones; egg viability was not tested. When we analyzed the oviposition success, we considered the percentage of females that presented an oviposition value equal to or greater than the control median, while Petersen et al.^38^ considered individual females that laid at least one egg, in a qualitative analysis.

Our results regarding the success of oviposition indicated that CHIKV-infected females showed a small increase of about three percent in the oviposition efficiency when feeding was performed in the second GC. ZIKV-infected females, on the other hand, showed to be less efficient in this GC, showing a greater decrease in almost half of their efficiency percentage. Moreover, in relation to the percentage of null females, those infected with CHIKV presented infertility of 3.8% and 2.7%, when feeding was performed in the first or second gonotrophic cycles, respectively. However, interestingly, the females infected with ZIKV presented a percentage of 2.1% and 6.8%, respectively, indicated that infertility increases when they get older as expected.

Unlike what was reported by previous studies^30,31^, in which they used in the second GC the same females that had already had the first oviposition, we aimed to isolate the age factor from the possible drop in viral load over time^31,40^. That is why we performed infective blood feeding only in the first or in the second GC.

The temporal tropism of the virus was taken into consideration when we decided to perform the infective blood feeding, since the females lay eggs about 4 days after feeding. According to Sirisena et al.^31^ and Ryckebusch et al.^40^, after 4 days of infection, CHIKV and ZIKV have already disseminated over the mosquito’s body, although CHIKV does it more quickly. Vega-Rúa et al.^16^ showed that CHIKV of distinct genotypes disseminated in 80–100% of *Ae. albopictus* in only 3 days after oral challenge. Le Coupanec et al.^28^ described the quick distribution of CHIKV particles in *Ae. aegypti* body, more specifically in the midgut (MG) and salivary glands (SG). The virus was detected in the SG at 4-day post viral exposure, peaking by day 8. The replication kinetics of CHIKV is different from other viruses, such as ZIKV, which seems to disseminate to secondary tissues of the mosquito body in a slower manner, with the peaking at 10–14 days after oral challenge^41,42^.

Finally, we observed a positive correlation between the number of laid eggs and the tendency of hatching viable larvae in younger females. Interestingly, CHIKV and ZIKV infection does not change this pattern in *Ae. aegypti*. On the other hand, when females are older, this correlation is lost in CHIKV infected females.

Our data revealed that ZIKV infection may increase infertility as the females get old but did not affect the viability of the eggs, while CHIKV infection affects viability and shows a loss of correlation of viability versus number of eggs. It indicates that a high oviposition efficiency (high number of eggs) does not correspond to a high number of viable larvae. CHIKV and ZIKV affects differently *Ae. aegypti* physiology, which can have relation with the different viral spread in nature. Understanding these parameters of vectorial capacity is crucial to elucidate the arboviruses transmission as well as the infected *Ae. aegypti* biology.

## Methods

### Mosquito rearing

*Aedes aegypti* mosquito eggs (PAEA strain from Tahiti, French Polynesia^43^, maintained in laboratory since 2003) were hatched in plastic trays containing 1.5 L of Milli-RO water and approximately 1 g of yeast (Vitalab, Brazil). First instar larvae were counted and redistributed to new plastic trays (300 larvae per tray) and fed with the same quantity of yeast, every 2 days, until pupae development (according to Farnesi et al.^39^. Pupae were counted and separated in cages (with approximately 400 each) for adult emergence; males and female mosquitoes were kept together (with 10% sucrose solution ad libitum) to allow copulation. For all experiments, mosquitoes were maintained in an incubator (Forlab Scientific Incubator, USA) at 25 ± 1 ºC, with a photoperiod of 12 h of light and dark (LD 12:12) and 60–80% relative humidity (RH).

### Virus and mosquito oral infection

The ZIKV strain ZIKV/H.sapiens/Brazil/PE243/201 (GenBank accession number KX197192.1) and CHIKV strain BHI3745/H804709^4^ were used for oral experimental infection of *Ae. aegypti* females.

These strains were harvested in C6/36 monolayer cells flask for 7 days, in Leibovitz-15 media supplemented with 5% fetal bovine serum, triptose 2.9 g/L, 0.075% sodium bicarbonate, 0.02% l-glutamine, 1% of non-essential amino acids and 1% penicillin/streptomycin at 28 °C according to Oliveira et al.^44^. Cell culture supernatant was collected and centrifuged at 1500*g* for 5 min. The aliquots were kept frozen − 70ºC until use.

Both viral titles were determined by plaque assay in Vero cells following 10 × serial viral stock dilution and covered by a layer of DMEM media supplemented with 2% fetal bovine serum, 1% penicillin/streptomycin and 0.8% methylcellulose, incubated for 3 days at 37 ºC and 5% CO_2_.

The mosquitoes were orally infected with seven (‘young females’) or fourteen (‘old females’) days old by blood meal containing 10^7^ PFU/ml of CHIKV or ZIKV. The infectious blood meal was prepared by mixing 1:1 of rabbit red blood cells and virus stock and 10% of heat—inactivated rabbit plasma. The mosquitoes were artificially fed using glass artificial feeders, sealed with Parafilm-M membrane stretched, connected to a bath at 37 °C for approximately 40 min, inside a Biosafety level—2 (BSL-2) insectary. Control mosquitoes fed on a similar blood meal, but with a non-infected L15 culture medium^44^. Prior to blood meals, female mosquitoes were deprived of sucrose for approximately 6 h. After blood meal, mosquitoes were cold anesthetized and only the fully engorged females were used.

### Viral confirmation

Total RNA from the whole mosquitoes was extracted individually, 4 days after virus infection, using TRIzol (Life Technologies) according to manufacturer's protocol. Viral RNA detection and quantification of Zika and Chikungunya were carried out through RT-qPCR with TaqMan Fast Virus 1-Step Master Mix Kit (Invitrogen, Carlsbad, CA, USA) in QuantStudio 6 Flex Real-Time PCR System (Applied Biosystems, Foster City, CA, USA). For ZIKV detection, reaction was conducted using 0.6 µM forward primer—5′-CTTGGAGTGCTTGTGATT-3′, genome position 3451–3468; 0.6 µM reverse primer—5′-CTCCTCCAGTGTTCATTT-3′, genome position 3637–3620 and 0.8 µM probe—5′FAM-AGAAGAGAATGACCACAAAGATCA-3′TAMRA, genome position 3494–3517. For CHIKV detection, reaction was conducted using 1.1 µM forward primer 5′-TCACTCCCTGTTGGACTTGATAGA-3, genome position 6856–6879; 1.1 µM reverse primer—5′-TTGACGAACAGAGTTAGGAACATACC-3, genome position 6981–6956), and 0.2 µM probe 5´FAM-AGGTACGCGCTTCAAGTTCGGCG-3´, genome position 919–6941^45,46^. Cycling conditions for reactions were the same, as follows: 50 °C for 5 min, 95 ºC for 20 s, followed by 40 amplification cycles of 95 °C for 15 s and 60 °C for 60 s.

### Gonotrophic cycle assays for fecundity and fertility

For young females assays we used eggs of the first GC. Seven days-old *Ae. aegypti* females were provided with infected or uninfected blood meal as previously described. Oviposition was stimulated 4 days after blood meal as described in Farnesi et al.^39^. About 30 females per condition, in each experiment, were individually isolated in an inverted plastic Petri dish (90 mm in diameter) with wet filter paper (Whatman No. 1) lining the lid. All females were allowed to oviposit for 90 min inside incubators (25 °C), in dark and 60–80% relative humidity conditions. After oviposition, females were released in cages, and randomly separated for posterior viral detection (at least five females per condition). Eggs were stored inside the incubators in a regimen of 12 h of light followed by 12 h of dark (L/D) until the end of embryogenesis.

For old females’ assays, the eggs of the first GC of 7-days-old females were discarded as described by Padilha et al.^30^. After that, females returned to the cages provided with sucrose 10% ad libitum until they were 14 days-old when they were fed with infected or uninfected blood meal. Then, they were individualized and stimulated to lay eggs of the second GC as described above. Eggs from second GC were used to the analyses. The eggs dried for 7 days inside incubators (LD12:12; 25 °C and 60–80% RH) and then were counted and tested for viability.

### Fertility assays

All the eggs obtained from individual females were tested for viability. Briefly, to stimulate hatching 50 mL of industrial yeast extract solution 0.15% (weight/volume) were added in each Petri dish placed in incubators for 24 h (25 °C, 60–80% RH and photoperiod LD 12:12), according to Farnesi et al.^39^. In general, the assays were made three times independently.

### Oviposition efficiency

To evaluate if infection with CHIKV or ZIKV interferes in the efficiency of females to lay eggs, we assumed they were efficient when they could lay a number of eggs equal or higher than the control median, considering the control group of each age and each infection condition. The median of all controls is near to the average of egg laid described to *Ae. aegypti* species^19,20^.

### Statistical analysis

At first, all physiological data were tested for normality by Shapiro–Wilk test. We applied Mann–Whitney test for fecundity and fertility, as described in each graphic legends. For correlation analysis between fecundity and fertility, was used the Spearman coefficient. All statistical assays were executed using GraphPad Prism 5 (GraphPad Software, San Diego, California, EUA) and *p* value < 0.05 was considered for significant differences.

### Ethical statement

All experiments carried out on this study were approved by the institutional Research Ethics Committees IOC/FIOCRUZ #LW34/14 and CEUA-UFRJ 149/19 (for use of rabbit blood). All experiments were performed in accordance with relevant guidelines and regulations.

## Data Availability

The data generated and analyzed during the current study are available upon reasonable request to the corresponding author.
